# Does Calomel Really Expel the Biliary Secretion?

**Published:** 1849-03

**Authors:** 


### MATERIA MEDICA AND THERAPEUTICS.

*Does Calomel really Expel the Biliary Secretion?*—Dr. Michea has published, in *L'Union Médicale,* a very interesting paper on the above question. The author's object was to ascertain, by chemical analysis, whether the green colour which purgative doses of the chloride of mercury give to the alvine dejections (besides rendering the latter more copious and less dense) is really owing to a superabundant secretion of bile. Opinions, says Dr. Michea, are not agreed on this point either in France, Germany, or England. M. Higgins (who published his paper in *L'Union Médicale*) and M. Mialhe consider that calomel really excites the biliary secretion. MM. Trousseau and Pidoux, authors of an esteemed work on materia medica, express their doubts on the point. Actual experiments have been made by Dr. Franz Simon, Dr. Golding Bird, and M. Siebert. The first of these inquirers found, after large doses of calomel, a great quantity of bile and biliverdine; the second discovered only a few traces with a hydrocephalic child taking mercury, and the third maintains that the alvine dejections following the use of this metal present no trace whatsoever of bile. The green stools resulting from the use of the Carlsbad and Marienbad waters are, on the other hand, denied by M. Kerstin, of Freiberg, to contain any trace of bile, and that physician thinks the colour to be due to green sulphuret of iron, by the reduction, in the stomach and intestines, of the sulphate of soda contained in mineral waters, into a sulphuret, which subsequently combines with the iron likewise to be found in these waters. This theory is founded upon the fact, that hydrochloric acid removes the green colour of the fæces, and evolves a large amount of sulphuretted hydrogen. Dr. Golding Bird and Professor Schönlein are of opinion that the green colour given to alvine dejections by calomel is due, not to an excess of bile, but to an alteration of the hæmatosine. Startled by these dissimilar statements, Dr. Michea began a series of chemical analyses upon—1, the spontaneous alvine dejections of healthy men; 2, the same substance, of a more or less green colour, from men affected with gastro-intestinal imflammation; 3, the same, resulting from various doses of calomel; and 4, evacuations produced by neutral salts and resinous purgatives. The author prefers for his tests, the strong nitric acid of Dumas to the sulphuric acid and syrup of Pettenkoffer. The spontaneous alvine dejections of six healthy individuals, four adults, and three children, were examined; their filtered solution remained unaltered by nitric acid. The evacuations of three patients affected with gastro-intestinal derangement were examined, and much bile was found in one case only. When the vomiting had subsided, the bile disappeared from the dejections. Calomel given to eight persons, five men and three women, in doses varying from twelve to fifteen grains, produced green stools in four patients only. These being analysed, it was found that they contained a superabundance of bile, and that, with nitric acid, two principles of that secretion might be made manifest,—viz., biliverdine and albumen. The evacuations of two of these subjects gave, not a pure green by nitric acid, as this reagent will generally produce on biliverdine, but a dirty olive, (on this Dr. Michea grounds his belief, that he found bile, and not biliverdine alone;) this olive colour, however, assumed the same successive shades of purple, red, and yellow, which biliverdine will yield. In the two other instances, the nitric acid gave a drab or yellowish-red colour, almost without any subsequent shades. The author puts the question, whether this might not have been the bilifulvine of Mülder. The evacuations of five persons who took neutral salts and resinous purgatives were never green, and exhibited no albumen on the addition of either by nitric acid or heat, whereas the albumen, as shown by a plentiful precipitate, was abundant with the four patients using calomel. This albumen was, according to Dr. Michea, furnished by the bile. These experiments would, then, tend to elucidate the practice,—First of English physicians, who regard calomel as a specific in liver affections; secondly, of Dr. Schönlein, in typhus, who looks for green evacuations by fifteen-grain doses of the chloride of mercury; and thirdly, of Russian practitioners, who consider calomel the most efficient agent against cholera. Modern organic chemists look upon bile as partly of an excrementitial nature, and that the liver as well as the lungs removes from venous blood substances which have become unfit for assimilation, (the resin and fat to be found in the bile containing much carbon and hydrogen.) The more plentiful, therefore, the secretion of bile, the purer the blood. Thus it becomes clear how calomel may act beneficially in miasmatic contaminations, in typhus and cholera. We subjoin Dr. Michea's conclusions:—

1. Calomel acts in a special and direct manner on the liver: this salt occasions alvine evacuations of a peculiar colour, due to an excess of actual bile, as shown by the action of nitric acid, which points to the presence of its colouring matter (biliverdine) by change of colouration, and of its albumen by precipitating the latter.

2. This influence of calomel upon the biliary secretion is not constant. It varies according to certain conditions and circumstances.

3. The green evacuations produced by calomel are more frequent with men than women. (This the author supposes to be owing to the greater quantity of alkaline chlorides generated in the stomachs of men, which chlorides, according to Mialhe, would contribute to transform the chloride of mercury into a bichloride.)

4. These evacuations have a peculiar consistence—viz., a viscous liquidity, somewhat like oil, or white of eggs beaten up together.

5. In some affections of the intestinal canal, an excess of bile, to be detected by reagents, may be found in the evacuations.

6. Spontaneous alvine evacuations in healthy people are quite free from an excess of bile.

7. Neutral salts and resinous purgatives exercise no direct or special influence on the liver. The alvine dejections which they produce contain no excess of bile, remaining unaltered by nitric acid or heat.—*London Lancet.*

